# Association between Chronic Hepatitis B/C and Incidence of Osteoporosis and Bone Fractures: Results from a Retrospective Cohort Study

**DOI:** 10.3390/jcm13206152

**Published:** 2024-10-16

**Authors:** Sven H. Loosen, Alexander Killer, Hans Henrich Bock, Tom Luedde, Christoph Roderburg, Karel Kostev

**Affiliations:** 1 Department of Gastroenterology, Hepatology and Infectious Diseases, University Hospital Düsseldorf, Medical Faculty of Heinrich Heine University Düsseldorf, 40225 Düsseldorf, Germany; hans.bock@med.uni-duesseldorf.de (H.H.B.); tom.luedde@med.uni-duesseldorf.de (T.L.); christoph.roderburg@med.uni-duesseldorf.de (C.R.); 2 Epidemiology, IQVIA, 60549 Frankfurt, Germany

**Keywords:** viral hepatitis, HBV, HCV, CHC, CHB, osteopenia, osteoporosis, virus, infection, regression

## Abstract

**Background:** Osteoporosis and bone fractures affect health and quality of life. Since bone disease is multifactorial, identifying risk factors is key in prevention. There are multiple reports on how viral hepatitis, especially chronic hepatitis B (CHB) and chronic hepatitis C (CHC), are affecting bone disease, but results vary. Here, we analyzed the potential association between CHB/CHC and osteoporosis or bone fractures in a large outpatient cohort in Germany. **Methods:** We included 3136 outpatients with CHB and 15,608 matched non-hepatitis individuals as well as 2867 outpatients with CHC and 14,335 matched non-hepatitis individuals from the Disease Analyzer Database between 2005 and 2022. The main outcome was the 5-year cumulative incidence of osteoporosis and bone fractures as a function of either CHB or CHC. **Results:** Within 5 years of the index date, 2.9% vs. 1.6% of patients with and without CHB were diagnosed with osteoporosis (*p* = 0.001) and 1.0% vs. 0.4% were diagnosed with bone fractures (*p* < 0.001). Moreover, 3.3% of CHC patients and 2.2% of individuals without hepatitis C were diagnosed with osteoporosis (*p* = 0.002). In Cox regression analyses, CHB was significantly associated with an increased risk for osteoporosis (HR: 1.76) and fractures (HR:2.43) and CHC with osteoporosis (HR: 1.54). For both CHB and CHC, the association with osteoporosis was restricted to the female subgroup. **Conclusions:** CHB and CHC are associated with osteoporosis in women. CHB in male patients is associated with a higher risk of fractures. More research is needed to understand the underlying pathophysiological mechanisms.

## 1. Introduction

Osteoporosis is a major risk factor for fractures, reduced quality of life and mortality [1,2]. Due to its high prevalence in older societies, it is already a disease of major importance and cases are expected to increase further in the future [3]. Therefore, the assessment of risk factors for osteoporosis in terms of prevention and screening is of key importance to reduce the further increase in disease burden. The main risk factors for osteoporosis are female gender and age [4], while the modifiable factors are smoking, BMI, physical activity and diet, especially calcium and vitamin D intake [5]. Osteoporosis supposedly affects 22 million women and 5.5 million men in the European union [6] and 800,000 fractures occur in Germany per year [7].

As we know from the gastroenterologist’s point of view, bone density is linked to liver health [8]. In patients with liver disease, bone disease is particularly common in the cirrhosis stage but can also occur at earlier stages. On the other hand, there is growing evidence that sarcopenia in patients with liver cirrhosis is a major factor for mortality, so immobility caused by fractures should be avoided [9], as this can lead to a vicious circle for patients with osteoporosis [10].

In addition, there is growing interest in the interaction between bone and liver, known as the liver–bone axis [11]. As the liver plays a central role in metabolism and hormonal status, the effects of liver disease on osteoporosis are being investigated based on changes in individual proteins/hormones produced by the liver. The proteins/hormones involved in communication between the liver and bone growth and whose serum levels are affected by liver disease include, for example, insulin-like growth factor 1, fibroblast growth factor 21 and lecithin–cholesterol acryltransferase [12]. As a result of impaired liver function, the reported prevalence of osteoporosis in cirrhotic patients is very high at 20 to 50% [13]. For viral hepatitis, chronic inflammation, in particular, is considered to be responsible for osteoclast activation regardless of the stage of liver fibrosis/cirrhosis [11].

One of the main causes of liver disease worldwide is viral hepatitis, primarily the hepatitis B virus (HBV) and the hepatitis C virus (HCV) [14]. Hepatitis B is transmitted during birth (vertical transmission), sexual contact and blood contact. While 90% of infections are acute, around 10% of infections lead to a chronic course defined as remaining positive for hepatitis B virus for six months [15,16]. Patients with chronic hepatitis and elevated transaminases, liver fibrosis/cirrhosis, high viral load and pregnancy or immune-suppressive treatments are treated with nucleos(ti)id analogs to prevent disease progression [15]. Hepatitis C is mostly transmitted by blood via drug use or in health care settings or by sexual contact and leads to a chronic course in 80% of patients [17], also defined as six months of infection [18]. Viral hepatitis also remains an important cause of liver disease in Europe, albeit with a decreasing trend [19]. In Germany, 0.2–0.7% of the general population are HBsAg-positive (meaning having active hepatitis B infection), 0.2–1.9% are anti-HCV-positive and 0.2–0.4% are HCV RNA-positive [20].

We know about associations between HBV and HCV and developing osteoporosis and fractures [21,22,23,24,25], but there are varying results in terms of the strength of association. With regard to HBV and osteoporosis, there is also debate as to whether antiviral treatment, particularly with tenofovir disoproxil fumarate (TDF), promotes the development of osteoporosis compared to other antiviral drugs or patients without treatment [24,26,27].

As HCV treatment has improved dramatically since the introduction of direct antiviral drugs and most of the data we have come across on viral hepatitis and bone disease are from the 2010s, we wanted to re-examine the relationship between viral hepatitis and osteoporosis/fractures in the era of modern antiviral therapy in a high-income country.

## 2. Patients and Methods

### 2.1. Database

This study was based on the Disease Analyzer database (IQVIA), which contains data on drug prescriptions, diagnoses and basic medical and demographic data obtained directly in an anonymous format from computer systems used in the practices of general practitioners and specialists [28]. The database covers approximately 2500 outpatient practices in Germany. It has previously been shown that the panel of practices included in the Disease Analyzer database is representative of general and specialized practices in Germany [28]. Finally, this database has already been used in previous studies focusing on hepatitis [29] and osteoporosis [30,31].

### 2.2. Study Population

**Figure 1:**
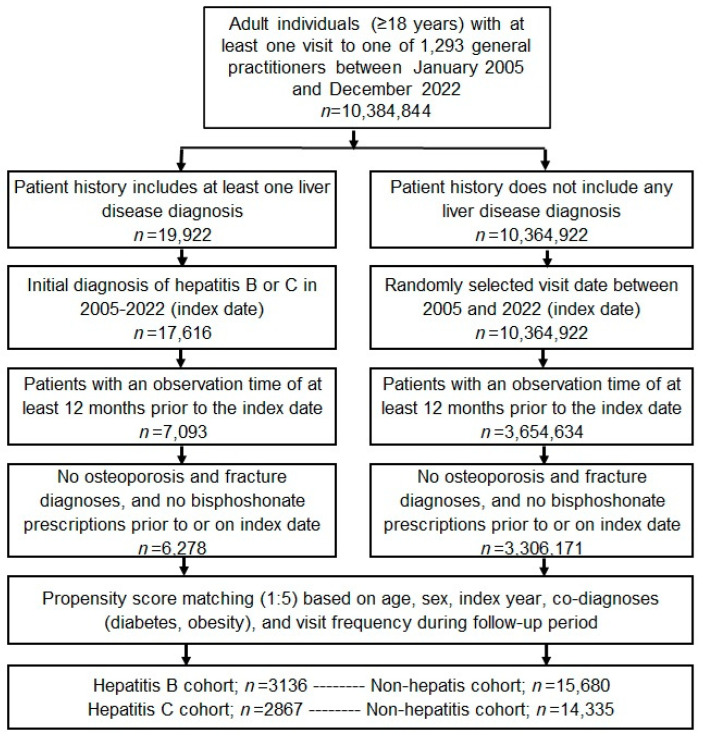
Selection of study patients.

This retrospective cohort study included adult patients (≥18 years) with an initial diagnosis of chronic hepatitis including chronic hepatitis B (CHB, ICD-10: B18.0, B18.1) and chronic hepatitis C (CHC, ICD-10: B18.2) from 1293 general practices in Germany between January 2005 and December 2022 (index date; Figure 1). Patients were only included when they had at least 12 months of pre-observation time prior to the index date. Patients with a diagnosis of osteoporosis (ICD-10: M80, M81) or a documented bone fracture (ICD-10: S02, S12, S22, S32, S42, S52, S62, S72, S82, S92, T02, T08, T10, T12) prior to or at the index date were excluded. Hepatitis patients were matched to individuals without a history of liver diseases by nearest-neighbor propensity scores based on sex, age, index year, obesity, diabetes and yearly consultation frequency. Diabetes and obesity were used as they are associated with osteoporosis and fractures. As hepatitis patients have a much higher GP consultation frequency, and higher consultation frequency can increase the probability of other diagnoses, we also included the consultation frequency per year in the propensity matching. For the non-hepatitis cohort, the index date was that of a randomly selected visit between January 2005 and December 2022 (Figure 1). Each kind of hepatis was compared with accordingly matched non-hepatitis individuals. Cohorts were considered to be adequately balanced if the absolute value of the standardized mean difference for each covariate was 0.25 or less.

### 2.3. Study Outcomes and Covariates

The main outcome of the study was the 5-year cumulative incidence of osteoporosis and bone fractures as a function of either chronic hepatitis B or C. As we were unable to differentiate between osteoporotic and non-osteoporotic fractures, we only consider fractures in patients who were also diagnosed with osteoporosis.

### 2.4. Statistical Analyses

Differences in the sample characteristics between patients with and without chronic hepatitis were tested using the Wilcoxon signed-rank test for continuous variables, the McNemar test for categorical variables with two categories and the Stuart–Maxwell test for categorical variables with more than two categories. The cumulative incidence of osteoporosis and fractures was evaluated using Kaplan–Meier curves. Cox regression models were conducted to study the association between CHB or CHC and the incidence of osteoporosis and/or bone fractures. Regression analyses were performed separately for women and men. No age-stratified analyses were conducted as the proportion of osteoporosis diagnosis in the age group < 50 years was too small for subgroup analysis. To counteract the problem of multiple comparisons (8 regression models), a *p*-value < 0.005 was considered statistically significant. Analyses were carried out using SAS version 9.4 (SAS institute, Cary, NC, USA).

## 3. Results

### 3.1. Baseline Characteristics

The present study included 3136 patients with CHB and 15,608 matched non-hepatitis individuals. Moreover, in a second cohort, we included 2867 patients with CHC and 14,335 matched non-hepatitis individuals. The baseline characteristics of the study cohort are shown in Table 1. The mean age [SD] was 50.2 [14.6] years in the CHB cohort and 50.8 [14.6] in the CHC cohort. A total of 52.7% of patients in the CHB cohort and 60.7% of patients in the CHC cohort were female. Due to the matched pair study design, no significant differences between variables were observed between the two hepatitis cohorts and their matched non-hepatitis cohorts.

### 3.2. Association of CHB and a Subsequent Diagnosis of Osteoporosis and Bone Fractures

**Figure 2:**
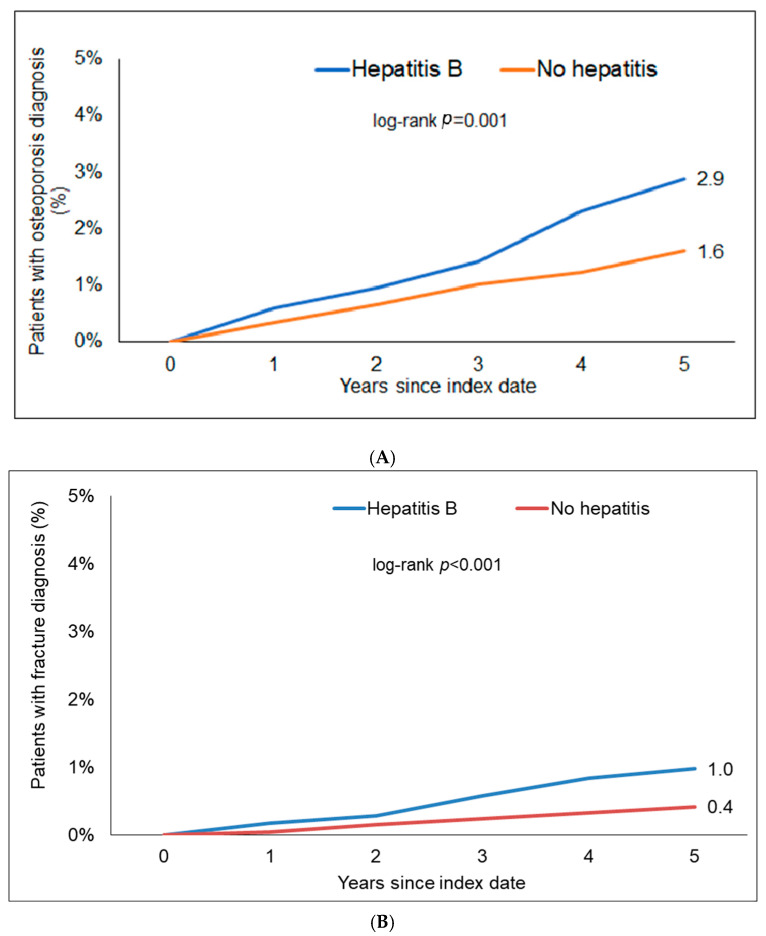
Kaplan–Meier curves showing the incidence of osteoporosis (**A**) and bone fractures (**B**) between patients with and without chronic HBV.

Within 5 years of the index date, 2.9% of CHB patients and 1.6% of individuals without hepatitis were diagnosed with osteoporosis (log-rank, *p* = 0.001). In terms of bone fractures, 1.0% of the CHB patients but only 0.4% of the hon-hepatitis patients were diagnosed with bone fractures (log-rank, *p* < 0.001, Figure 2). The most frequent fractures were shoulder and arm (33.9% in CHB, 34.1% in CHC and 33.9% in those without hepatitis), followed by foot and leg (23.4% in CHB, 22.5% in CHC and 27.6% in those without hepatitis) and ribs (15.6% in CHB, 19.1% in CHC and 17.0% in those without hepatitis).

In Cox regression analyses, CHB was significantly associated with an increased risk of osteoporosis (hazard ratio (HR): 1.76; 95% CI: 1.31–2.36) as well as bone fractures (HR: 2.43; 95% CI: 1.42–4.14). The association between CHB and osteoporosis reached the predefined level of statistical significance in women only (HR: 1.92; 95% CI: 1.38–2.69). Contrarily, the association between CHB and bone fractures was only significant in men (HR: 3.42; 95% CI: 1.44–8.12).

### 3.3. Association of CHC and a Subsequent Diagnosis of Osteoporosis and Bone Fractures

**Figure 3:**
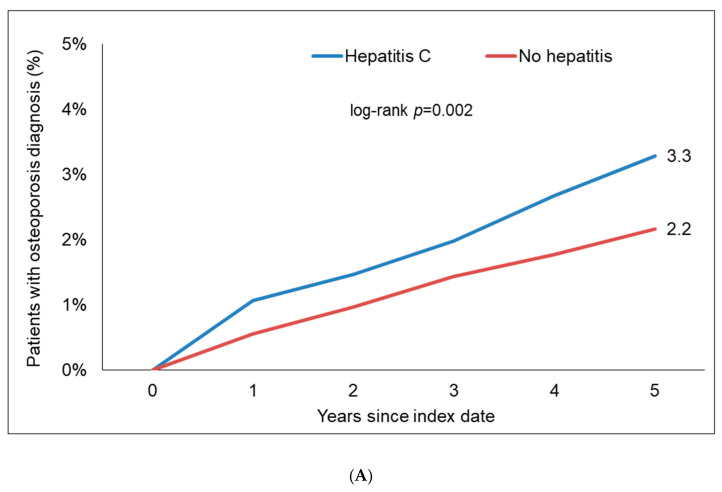

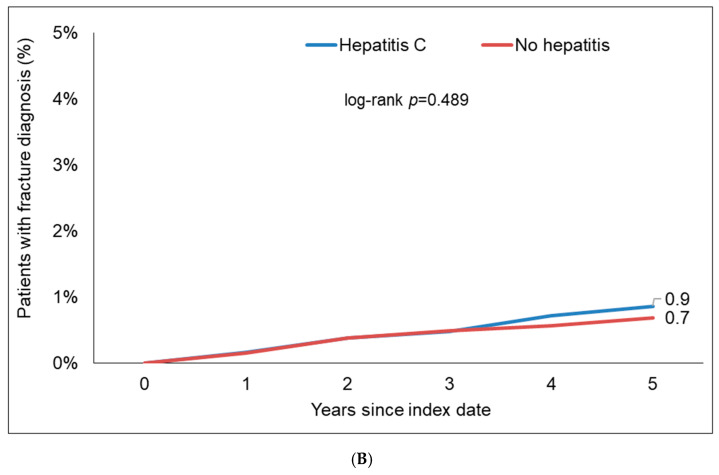
Kaplan–Meier curves showing the incidence of osteoporosis (**A**) and bone fractures (**B**) between patients with and without chronic HCV.

Within 5 years of the index date, 3.3% of CHC patients and 2.2% of individuals without hepatitis were diagnosed with osteoporosis (log-rank, *p* = 0.002). Moreover, 0.9% of CHC patients and 0.7% of the hon-hepatitis patients were diagnosed with a bone fracture (log-rank, *p* = 0.489, Figure 2). In regression analyses, CHC was significantly associated with an increased risk of osteoporosis (HR: 1.54; 95% CI: 1.17–2.02, Figure 3). In contrast, CHC was not associated with an increased risk of bone fractures (HR: 1.21; 95% CI: 0.71–2.06, Figure 3). Interestingly, the association between CHC and osteoporosis was only significant in women (HR: 1.75; 95% CI: 1.29–2.37) but not men (Table 2).

## 4. Discussion

In the present study, we evaluate a potential association between CHB and CHC and a subsequent diagnosis of osteoporosis or bone fractures in a large real-world cohort of CHC/CHB patients in Germany. We observe that CHB was significantly associated with an increased risk of osteoporosis and fractures and CHC with osteoporosis. Thereby, we confirm previous findings that CHB is associated with osteoporosis. A Korean study from 2019 compared the odds ratio for diagnosis of CHB or CHC in patients with osteoporosis and a matched control group [25]. Similar to our data, the association between CHB and osteoporosis was stronger than between CHC and osteoporosis. Furthermore, as in our data, the association was limited to the female subgroup. Tao et al. analyzed data from the US NHANES database for association of HBsAg-positive and HCV RNA-positive patients and lower bone mineral density and found positive relations for both [22]. A meta-analysis of 15 studies regarding HIV/HCV-coinfected patients showed a HR of 1.63 for coinfected patients compared to HIV mono-infected patients for the diagnosis of osteoporosis [32].

Our data show differing effects of viral hepatitis and sex. The HR for osteoporosis was 1.92 in women with CHB and 1.75 in those with CHC, while in men, there was no statistically significant association. This is somewhat counterintuitive, as CHB is known to affect liver health more in men than women. Men are more likely to develop HCC, less likely to clear HBeAg and less likely to be protected by HBV vaccination [33]. For HCV infection, there are also known gender differences: Women are more likely to clear HCV infections and progress slower towards cirrhosis [34]. Analysis of immune response and cytokine markers may help to explain the different effect on bone disease. Since our analysis did not include any immune or inflammatory markers, we encourage further research on this matter. Primary osteoporosis is in general more prevalent in women than in men, while secondary osteoporosis is in general more frequent in men, which is contrary to our result [35].

The significant higher hazard ratio/prevalence of osteoporosis in female patients could lead to larger patient samples for that subgroup and explain why differences are only statistically significant for women.

Of note, the association between CHB and fractures was only significant in men with an HR of 3.42 in the reported data of our study. In contrast, we could only find a non-significant association between CHB and osteoporosis in men. Therefore, it seems likely that fractures were not related to osteoporosis and probably more frequent in male CHB patients because of confounders that were not analyzed. For example, one confounder leading to more fracture risk could be alcohol consumption, which is also reported to be an important factor for patients with HBV infection in terms of liver disease progression [36,37]. In conclusion, we did find significant differences between sexes but a lack of more data prevents us from explaining the differences.

As a clinical consequence of our research and already published data, patients with chronic HBV and/or HCV should be screened for risk of osteoporosis and this risk should be addressed in patient communication. Following recommendations for osteoporosis screening, bone mineral density testing should be carried out for all patients aged 50 with risk factors for fracture/osteoporisis [38]. Diagnosis of osteoporosis and the following treatment reduces the risk for following fractures underlining the importance of screening [38].

Lifestyle factors like smoking or physical exercise should be optimized, since they do not only affect risk for osteoporosis but also risk of liver disease progression. In particular, the importance of alcohol intake in progression to cirrhosis and its negative impact on bone health should be emphasized [39,40]. Patients should also be evaluated for vitamin D supplementation and/or guided for improvement of diet choices. Also, comedication that can worsen development of osteoporosis or fractures such as proton pump inhibitors [41] or benzodiazepines [42], for example, should be evaluated for cessation.

By study design, this study is limited because of its retrospective nature. Furthermore, we cannot differentiate between treated and untreated patients, meaning we can only report associations between diagnosis of viral hepatitis and osteoporosis or fractures. Since antiviral treatment of HBV reportedly affects risk of osteoporosis, this is further limiting. A major limit for analysis of HCV-positive patients is that we cannot differentiate between cured and not cured patients. We also cannot match lifestyle factors such as physical exercise, BMI, alcohol intake or smoking. Smoking is, for example, more frequent in HCV patients than in the general population [43,44]. In an American study, hepatitis C-positive people were almost three times more likely to smoke than hepatitis C-negative people [45]. Finally, sufficient information on the presence of liver cirrhosis was not available, so we were unable to differentiate between patients with and without liver cirrhosis.

## 5. Conclusions

Our study supports prior data on the association between chronic viral hepatitis and development of osteoporosis. We report differences between female and male patients that need further research. Also, changes in risk for osteoporosis after finite antiviral treatment for hepatitis C should be evaluated. Hepatologists and general practionioners treating patients with viral hepatitis should take note of the osteoporosis risk and evaluate screening, prevention and treatment of osteoporosis.

## Figures and Tables

**Table 1:** Basic characteristics of the study sample (after 1:5 matching).

Variable	Proportion Affected among Patients with Chronic Hepatitis (N, %)	Proportion Affected among Patients without Chronic Hepatitis (N, %)	*p*-Value
**Hepatitis B**			
N	3136	15,680	
Age (Mean, SD)	50.2 (14.6)	50.1 (14.6)	0.945
Age ≤ 40	855 (27.3)	4278 (27.3)	0.995
Age 41–50	763 (24.3)	3799 (24.2)	
Age 51–60	757 (24.1)	3766 (24.0)	
Age > 60	761 (24.5)	3837 (24.5)	
Men (%)	1485 (47.3)	7394 (47.2)	0.840
Women (%)	1651 (52.7)	8286 (52.8)	
Diabetes	541 (17.3)	2685 (17.1)	0.863
Obesity	397 (12.7)	2004 (12.8)	0.853
Yearly consultation frequency	6.4 (4.1)	6.4 (4.1)	0.746
**Hepatitis C**			
N	2867	14,335	
Age (Mean, SD)	50.8 (14.6)	50.8 (14.6)	0.959
Age ≤ 40	766 (26.7)	3853 (26.9)	0.994
Age 41–50	737 (25.7)	3662 (25.6)	
Age 51–60	685 (23.9)	3.405 (23.7)	
Age > 60	679 (23.7)	3415 (23.8)	
Men (%)	1143 (39.9)	5706 (39.8)	0.950
Women (%)	1724 (60.1)	8629 (60.2)	
Diabetes	434 (15.2)	2158 (15.1)	0.909
Obesity	218 (7.6)	1074 (7.5)	0.836
Yearly consultation frequency	7.2 (4.5)	7.2 (4.5)	0.932

**Table 2:** Association between hepatitis B and C and the incident osteoporosis and fracture diagnoses in patients followed in general practices in Germany (Cox regression models).

	Hepatitis vs. No Hepatitis (HR, 95% CI)		
	Total	Women	Men
Hepatitis B versus no liver diseases			
Outcome: Incidence of osteoporosis	1.76 (1.31–2.36) *	1.92 (1.38–2.68) *	1.29 (0.70–2.40)
Outcome: Incidence of bone fractures	2.43 (1.42–4.14) *	1.95 (0.99–3.88)	3.42 (1.44–8.12) *
**Hepatitis C versus no liver diseases**			
Outcome: Incidence of osteoporosis	1.54 (1.17–2.02) *	1.75 (1.29–2.37) *	0.97 (0.51–1.86)
Outcome: Incidence of bone fractures	1.21 (0.71–2.06)	1.25 (0.69–2.26)	1.05 (0.30–3.69)

* *p* < 0.005.

## Data Availability

The underlying data are available upon reasonable request from the corresponding author.
